# Human Umbilical Cord Blood-Derived Mesenchymal Stem Cells Promote Vascular Growth *In Vivo*

**DOI:** 10.1371/journal.pone.0049447

**Published:** 2012-11-16

**Authors:** Santiago Roura, Juli R. Bagó, Carolina Soler-Botija, Josep M. Pujal, Carolina Gálvez-Montón, Cristina Prat-Vidal, Aida Llucià-Valldeperas, Jerónimo Blanco, Antoni Bayes-Genis

**Affiliations:** 1 ICREC Research Program, Fundació Institut dInvestigació en Ciències de la Salut Germans Trias i Pujol (IGTP), Badalona, Spain; 2 Cardiovascular Research Center, CSIC-ICCC, Barcelona, Spain; 3 Networking Biomedical Research Center on Bioengineering, Biomaterials and Nanomedicine (CIBER-BBN), Barcelona, Spain; 4 Cardiology Service, University hospital Germans Trias i Pujol, Badalona, Spain; 5 Department of Medicine, Universitat Autònoma de Barcelona, Barcelona, Spain; Brigham and Women's Hospital, United States of America

## Abstract

Stem cell therapies are promising strategies to regenerate human injured tissues, including ischemic myocardium. Here, we examined the acquisition of properties associated with vascular growth by human umbilical cord blood-derived mesenchymal stem cells (UCBMSCs), and whether they promoted vascular growth *in vivo*. UCBMSCs were induced in endothelial cell-specific growth medium (EGM-2) acquiring new cell markers, increased Ac-LDL uptake, and migratory capacity as assessed by qRT-PCR, Western blotting, indirect immunofluorescence, and invasion assays. Angiogenic and vasculogenic potentials could be anticipated by *in vitro* experiments showing self organization into Matrigel-mediated cell networks, and activation of circulating angiogenic-supportive myeloid cells. In mice, following subcutaneous co-injection with Matrigel, UCBMSCs modified to co-express bioluminescent (luciferases) and fluorescent proteins were demonstrated to participate in the formation of new microvasculature connected with the host circulatory system. Response of UCBMSCs to ischemia was explored in a mouse model of acute myocardial infarction (MI). UCBMSCs transplanted using a fibrin patch survived 4 weeks post-implantation and organized into CD31^+^network structures above the infarcted myocardium. MI-treated animals showed a reduced infarct scar and a larger vessel-occupied area in comparison with MI-control animals. Taken together, the presented results show that UCBMSCs can be induced *in vitro* to acquire angiogenic and vasculogenic properties and contribute to vascular growth *in vivo*.

## Introduction

Stem cell-based therapies are promising strategies to regenerate human injured tissues, including ischemic myocardium, by supporting new vascular growth and/or repair. Commonly, distinct mechanisms regulate blood vessel formation, including sprouting of mature endothelial cells (angiogenesis), recruitment of circulatory genuine endothelial progenitor cells and/or accessory pro-angiogenic cells (vasculogenesis), and internal division of pre-existing capillary plexus without sprouting (intussusceptive angiogenesis) [1], [2]. Molecularly, among others, vascular endothelial growth factor (VEGF) and stromal cell-derived factor (SDF)-1α trigger vascular development following activation of genes encoding early growth response factor-3 (Egr-3) [3]–[5], CD31 [6] and integrin-linked kinase (ILK) [7], [8].

To date, the acquisition of properties related to vascular growth has been reported for bone marrow progenitors [9]–[11], umbilical cord blood (UCB)-derived CD34^+^precursors [12] and, more recently, embryonic stem cells [13]. In addition, UCB contains mesenchymal stem cells (MSCs) that improve neurological function recovery through angiogenesis in rats [14]. However, both differentiation and contribution to functional blood vessel growth were poorly characterized and not irrefutably demonstrated *in vivo* [14].

Thus, in the present study, we further examined the acquisition of properties associated with vascular growth (including those of active migratory capacity and Matrigel-mediated cell network formation, as well as activating circulating angiogenic-supportive myeloid cells [15], [16]) by UCBMSCs *in vitro*. Whether UCBMSCs promoted vascular growth *in vivo* was then explored following subcutaneous co-injection with Matrigel and in an acute myocardial infarction (MI) model in mice.

## Results

### EGM-2-induced UCBMSCs Acquired Angiogenic Properties *in vitro*

**Figure 1:**
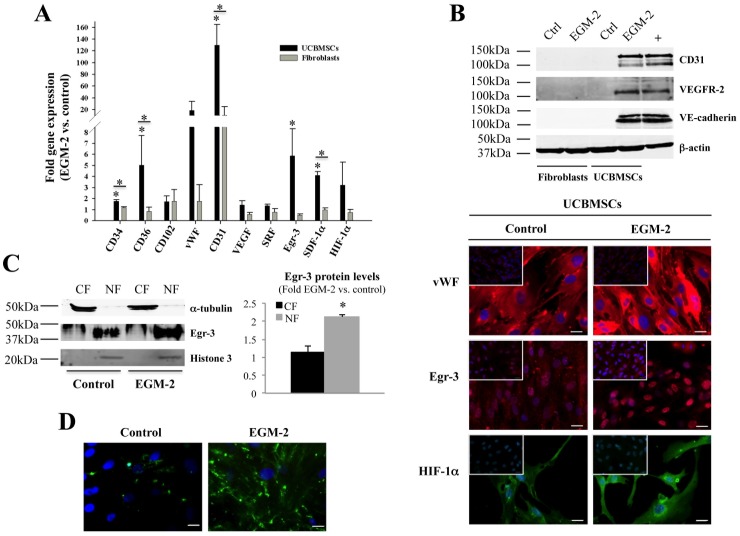
EGM-2 induces new phenotypic and functional properties in UCBMSCs. **A**) Comparative gene expression analysis of both UCBMSCs and fibroblasts expanded in control and EGM-2. **B**) Analysis of CD31, VEGF-R2 and VE-cadherin protein levels in both UCBMSCs and fibroblasts (50 µg of whole cell lysates) by Western blotting. Lane labelled with “+” corresponds to VEGF-treated HUVECs (20 µg). Detection of β-actin was carried out for internal protein loading control. Representative indirect immunofluorescence images showing vWF, Egr-3 and HIF-1α expression in UCBMSCs in control and EGM-2 conditions. Upper left inserts display immunostaining of fibroblast cultures under the same experimental conditions. Bars = 20 µm. **C**) Western blot analysis of Egr-3 protein levels in 50 µg of total cytoplasmic (CF) and nuclear (NF) fractions from control and EGM-2-cultured UCBMSCs. Histogram shows the densitometric quantification of Western blot data. α-tubulin and histone-3 were used as internal protein loading controls. **D**) Representative images showing differences in uptake of Alexa488-conjugated Ac-LDL by UCBMSCs in control and EGM-2 conditions. A minimum of 10 visual fields per immunofluorescence experiment (N = 3) were analyzed. Bars = 20 µm. *P<0.05 and A.U. = arbitrary units.

Primary cultures of elongated fibroblast-like cells were established from human UCB samples. Cultured cells were homogenously recognized as MSCs by two independent criteria (standard surface antigen pattern and multipotency). Importantly, flow cytometric analysis ensured that UCBMSC cultures were strictly homogeneous and did not show baseline expression of neither endothelial nor hematopoietic cell traits (**Figure S1A**). In order to determine whether properties associated with vascular growth could be also induced in UCBMSCs, cells were cultured in EGM-2 for 15 days and subjected to gene expression analysis by qRT-PCR. Our results showed significant increases in CD34, CD36, CD31, Egr-3, and SDF-1αgene activation (*P*<0.05), but the cell growth and differentiation modulators VEGF and serum response factor (SRF) were not differentially enhanced (**Figure 1A**). At protein level, induction of CD31, VEGFR-2, and VE-cadherin was corroborated by Western blotting (**Figure 1B**). Although not a quantitative procedure, indirect immunofluorescence was used to evidence that vWF, Egr-3, and HIF-1α protein levels tended to increase (**Figure 1B**). For Egr-3, further analysis of subcellular distribution showed both preferential location within cell nuclei and increased levels in induced UCBMSCs (**Figure 1C**). These responses to EGM-2 were not detected in primary human fibroblasts (**Figure 1A,B**).

Moreover, both increased uptake of Ac-LDL from the extracellular medium (**Figure 1D**) and surface expression of CD31 and CD34, consistent with those measured in primary cultures of bona fide ECs such as human dermal blood microvascular cells (used as positive controls) (**Figure S1B**), were also observed in induced UCBMSCs. Incubation with a purified monoclonal antibody against CD31 suppressed intercellular adhesion between induced UCBMSCs, indicating that *de novo* CD31 protein was functional (data not shown).

We next studied whether induced UCBMSCs showed the migratory behaviour characteristic of angiogenic cells. Thus, cell migration assays using Culture-Inserts showed that induced UCBMSCs exhibited a high capacity to colonize cell-free tissue culture surface (surface recovery index), and were able to close a “scratch wound” reaching confluence even faster than fibroblasts (used as positive migratory cells [17]) (**Figure S2A**). Furthermore, in order to ensure that an increase in cell proliferation activity was not responsible for the marked surface recovery index exhibited by induced cells in the “scratch wound” assay, cell proliferation curves were obtained for UCBMSCs in both control and EGM-2 conditions. Induced cells showed a significantly shorter doubling time in comparison with that of UCBMSCs in control medium (1.5±0.14 vs. 2.16±0.06 days; *P* = 0.009). In immunofluorescence experiments, higher levels of the nuclear Ki67 protein, an excellent marker to determine the growing fraction of a given cell population [18], were detected in UCBMSCs cultured in EGM-2 compared to untreated cell cultures, also indicative of faster proliferating cells (**Figure S2B**). The proliferation rate of induced UCBMSCs was in no case inferior to 24 hours, the time period taken by the “scratch wound” assay. In addition, migration of induced cells was accompanied by increases in CD31, CD36 and Egr-3 gene activation (300, 65 and 8-fold, respectively) in comparison with those found in cells remaining within Culture-Inserts (data not shown).

**Figure 2:**
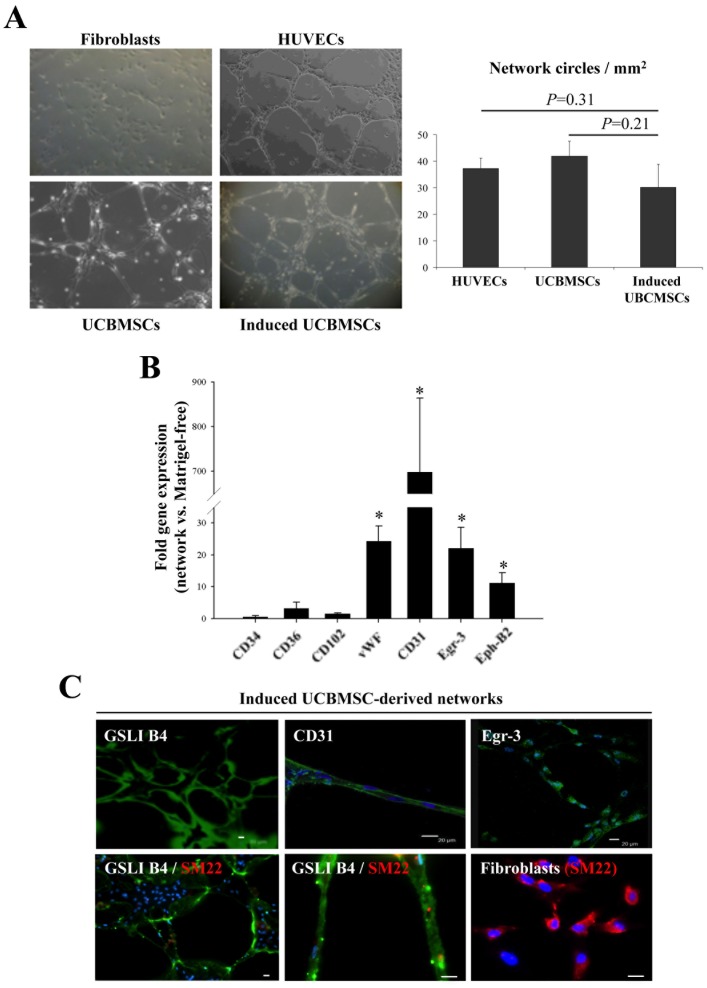
Matrigel-mediated induction of self-assembled cell networks by UCBMSCs. **A**) Representative images of cell networks generated by both control and EGM-2-induced UCBMSCs during 5 h in Matrigel. Primary fibroblasts and HUVECs were used as negative and positive controls, respectively. Histogram represents pro-angiogenic capacity measured as the number of regular network circles per mm^2^ from three independent experiments. **B**) Analysis of gene transcription by qRT-PCR comparing induced UCBMSC networks and the same cells in Matrigel-free conditions. **C**) Representative green-fluorescence images showing, top row (left to right): biotynilated GSLI B4 staining, specific detection of surface CD31, and nuclear Egr-3 in self-assembled cell networks developed by UCBMSCs; bottom row (left to right): GSLI B4 and SM22 staining of UCBMSC-derived network (left), higher magnification of previous image (middle), and SM22 staining in human fibroblasts (right). Nuclei were labelled blue with Hoechst. *P<0.05 and Bars = 20 µm.

Cell network-forming capacity in Matrigel, a standard but preliminary *in vitro* two-dimensional test of angiogenesis, was then assessed. Remarkably, control (uninduced) UCBMSCs aligned forming well-organized, branched networks after 6 hours, as also did umbilical vein endothelial cells (HUVECs) (*P* = 0.31), which were used as bona fide angiogenic cells, and induced UCBMSCs (*P* = 0.21) (**Figure 2A**). These results suggested a high cell network-forming potential by UCBMSCs, even in control conditions. Moreover, compared to Matrigel-free conditions, self organization into cell networks was accompanied by elevated transcription of genes encoding CD31 (698-fold), CD36 (3.2-fold), vWF (24.3-fold), Egr-3 (22.1-fold) and Ephrin-B2 (11.2-fold), a protein involved in the acquisition of vascular properties by MSCs [19], [20] (**Figure 2B**). High protein levels of extracellular membrane CD31 and nuclear Egr-3 were also detected in the cell networks developed by UCBMSCs following induction in EGM-2 (**Figure 2C**). The organized networks were also strongly positive for *Griffonia Simplicifolia* Lectin I (GSLI) B4 isolectin staining but no associated SM22^+^smooth muscle cells were detected (**Figure 2C**).

### Induced UCBMSCs Activated Circulating Myeloid Cells *in vitro*

**Figure 3:**
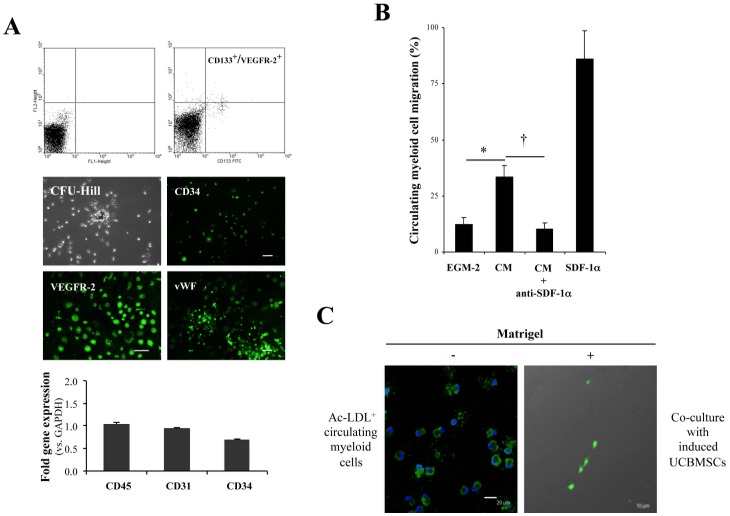
Activation of circulating myeloid cells by induced UCBMSCs. **A**) Characterization of myeloid (CD45^+^) cells derived from human peripheral venous blood and their progeny in cell culture. Representative flow cytometric dot blots showing co-expression of CD133 and VEGFR-2 in isolated cell fraction. Quadrants were first placed on FL1/FL2 dot plot based on negative control staining for the subsequent detection of dual labelled cells. Representative images showing a colony-forming unit-Hill grown over human fibronectin (top left) and detection of CD34 (top right), VEGFR-2 (bottom left) and vWF (bottom right) after 14 days in culture. Histogram shows the transcriptional levels of CD45, CD31 and CD34 genes in the extracted myeloid cell subset quantified by qRT-PCR. Data are expressed as mean ± S.D of Ct values and each value was normalized to the corresponding GAPDH value. N = 5 and bars = 20 µm. **B**) Histogram showing results from transwell chemotaxis assays used to quantify SDF-1α-mediated migration of circulating myeloid cells. Human recombinant SDF-1α was used as positive control. N = 3. *P = 0.02 and ^†^ *P* = 0.016. **C**) Representative images displaying Alexa488-Ac-LDL-labelled circulating myeloid cells in Matrigel-free conditions, and a representative ‘hybrid’ cell network comprising EGM-2-induced UCBMSCs and Alexa488-Ac-LDL^+^myeloid cells after co-culture in Matrigel during 7 h. Note that fluorescent, circulating myeloid cells with characteristic amoeboid morphology were not visible as single cells, but were closely associated with or aligned along the cell network developed by induced UCBMSCs. Bars = 20 µm (left) and 10 µm (right). A minimum of 10 microscopic fields per experiment (N = 3) were registered under a confocal microscope. CM = conditioned medium.

Prior to *in vivo* experimentation, the marked activation of SDF-1α gene expression found in induced UCBMSCs prompted us to further assess their capacity to activate peripheral venous blood-derived myeloid cells, specifically those selected by co-expression of CD133 and VEGFR-2, that develop adherent colonies (referred to as colony-forming unit-Hill [15]) when cultured in fibronectin-coated plates [21] (**Figure 3A**). The hematopoietic/myeloid origin of these colony-forming cells, which support vascular network formation *in vitro* and blood vessel growth *in vivo* [16], was confirmed by the analysis of CD45 gene expression by qRT-PCR (**Figure 3A**). Furthermore, the progeny of these circulatory pro-angiogenic cells was also characterized to express CD34, VEGFR-2, and vWF during culture *in vitro* (**Figure 3A**), as well as rarely self organize in Matrigel [22]. By performing transwell assays we established that conditioned medium collected from induced UCBMSCs, used as chemoattractant, increased migration of the isolated myeloid cells in comparison with fresh EGM-2 (*P* = 0.02). Moreover, this effect was suppressed by pre-incubation of the conditioned medium with a purified monoclonal antibody against SDF-1α (*P* = 0.016). This result was a clear indication that this well-known mobilizing cytokine [23] was responsible for the observed migratory behaviour of myeloid cells (**Figure 3B**). Moreover, in a Matrigel co-culture setting similar to that described in [16], using a 6∶1 (induced UCBMSC to myeloid cell) ratio, myeloid cells pre-labelled with Alexa488-Ac-LDL associated with or aligned along the networks developed by induced UCBMSCs (**Figure 3C**).

### Participation of UCBMSCs in Functional Microvascular Structures *in vivo*

Since uninduced UCBMSCs had previously demonstrated a high network-forming potential *in vitro*, we developed an experimental model to explore their ability to promote vascular growth *in vivo*. The model, in which we also expected to compare UCBMSCs and adipose tissue-derived progenitor cells (ATDPCs) (also a potential stem cell source for cardiovascular repair [24]), was based on the administration of cells modified to co-express luciferases (luc) and fluorescent proteins.

**Figure 4:**
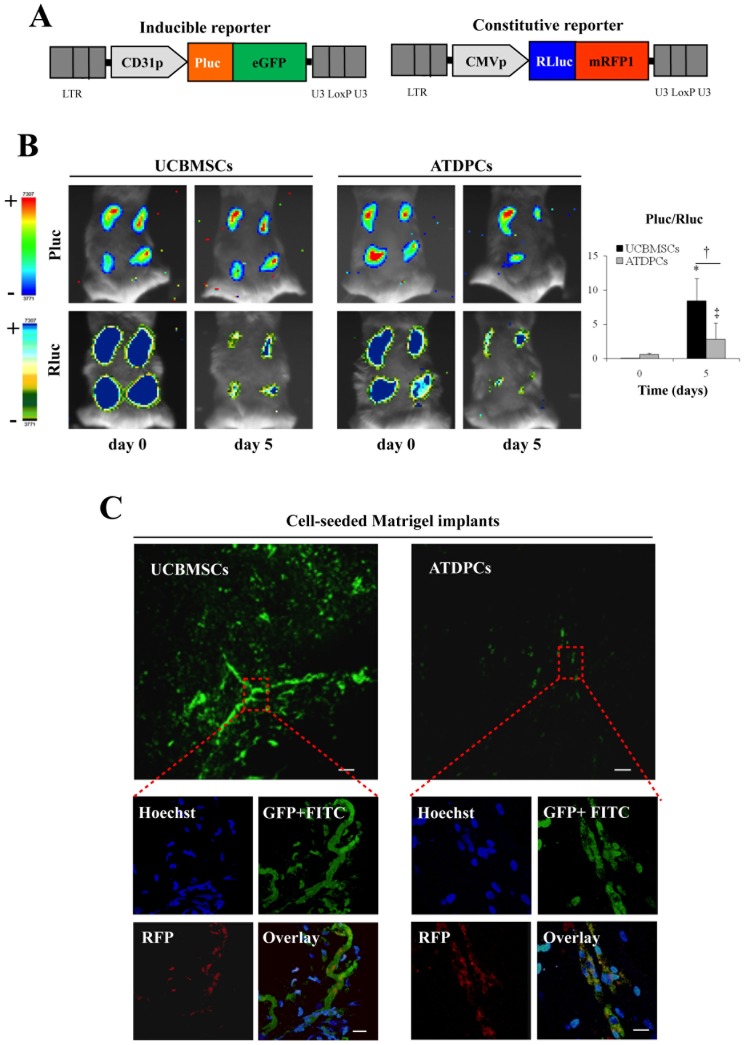
Participation of UCBMSCs in functional microvascular structures *in vivo*. **A**) Schematic representations of the inducible (CD31p-Pluc-eGFP) and constitutive (CMVp-Rluc-RFP) lentiviral vectors, in which each luciferase and fluorescent signal generated following promoter activation appears remarked with the corresponding colour. **B**) Representative registrations of Pluc and Rluc activities from dual labelled UCBMSCs and ATDPCs co-implanted with Matrigel superimposed on black white dorsal images of the recipient animal. Colour bars illustrating relative light intensities from Pluc and Rluc; low: blue and black; high: red and blue, respectively. Histogram represents over time quantification of implanted cell differentiation degree measured as the ratio between Pluc and Rluc signals. The region-of-interest (ROI) for the quantification of photon emission included only the individual injection sites. *P<0.001, ^†^ *P* = 0.032 and ^‡^ *P* = 0.047. **C**) Representative green-fluorescence images showing formation of microcirculatory vessels within UCBMSC- and ATDPC-seeded Matrigel implants removed from animals in which circulatory system was previously filled with FITC-dextran. Bars = 100 µm. Additional merged images show a functionally connected cell network better organized within Matrigel implants seeded with fluorescent labelled UCBMSCs than in those seeded with ATDPCs. ATDPCs appear more disperse and not well-organized. Bars = 20 µm.

UCBMSCs and ATDPCs were first transduced (∼70% efficiency) (data not shown) with the cytomegalovirus (CMV) promoter (p)-*Renilla reniformis* (R) luc-monomeric red fluorescent protein (mRFP1) lentiviral vector (**Figure 4A**). Subsequently, transduced cells selected by fluorescence-activated cell sorting (FACS) were transduced a second time, now with the CD31p-*Photinus pyralis* (P) luc-enhanced green fluorescent protein (eGFP) construct. Finally, cells were mixed with Matrigel and injected subcutaneously in four independent sites of the animal dorsal region.

With our strategy, bioluminescence imaging (BLI) allowed non-invasive monitoring of light production by CD31p- and CMVp-regulated Pluc and Rluc over time. In addition, the ratio between both activities measured the increase or decrease in CD31p-regulated expression relative to that derived from the internal standard CMVp. Previous *in vitro* experiments using co-transduced UCBMSC cultures to measure the relation between cell number and light production by Rluc, as well as the induction of CD31p-regulated Pluc during EGM-2 treatment were performed to validate this approach (**Figure S3**). Then, by confocal microscope analysis of bioluminescent UCBMSCs in culture and in implants from live mice, we showed that expression of both CD31p-regulated eGFP and CD31 was induced by EGM-2 (**Figure S4A**) and following co-injection with Matrigel (**Figure S4B**). Analysis of multiple image fields also showed that while eGFP^+^cells were undetectable in uninduced cell cultures (**Figure S4A**), 73% of the cells were simultaneously mRFP1^+^/eGFP^+^and expressed CD31, 23% were only mRFP1 positive, and 4% were only eGFP positive following EGM-2 induction (data not shown). Taken together, this analysis demonstrated that CMVp-Rluc-mRFP1 was not silenced. Moreover, cell cultures were highly homogeneous and no pre-differentiated cells could be detected before cell implantation.

BLI analysis of UCBMSC-seeded Matrigel implants in mice showed a large increase in the Pluc/Rluc ratio between days 0 and 5 post-implantation (0.05±0.03 vs. 8.4±3.2 times, respectively; *P*<0.001) that suggested a marked activation of the CD31p (**Figure 4B**). In addition, this activation was significantly higher in UCBMSC-seeded implants than in those seeded with ATDPCs (0.6±0.16 (day 0) vs. 2.8±2.4 (day 5) times) (*P* = 0.032) (**Figure 4B**). Counting of the three types of positive fluorescent cells, mRFP1^+^and eGFP^+^, only mRFP1^+^and only eGFP^+^showed that, at sacrifice, the proportion of eGFP^+^cells that were not also mRFP1^+^in the Matrigel plugs was extremely low (<1%) while 87% of the detected mRFP1^+^cells were also eGFP^+^(**Figure S4B**), confirming that a drop in the activity of CMVp-Rluc-mRFP1 was not the cause of the increase in the Pluc/Rluc ratio observed by BLI.

To validate BLI data with an independent procedure, Matrigel implants were dissected following the *in vivo* imaging period and analyzed by qRT-PCR. We found increases in human CD31 (378-fold), CD36 (77-fold), and vWF (2-fold) gene transcription in UCBMSC-seeded implants, supporting previous BLI data (**Table S1**). Interestingly, the pattern of activated genes included those encoding VEGF (81-fold) and HIF-1α (50-fold). Egr-3, ILK, EphB2, and SDF-1α gene transcription was also enhanced in UCBMSC-seeded implants (**Table S1**). Different patterns of gene activation in cell-seeded Matrigel implants between UCBMSCs and ATDPCs were detected. While in UCBMSCs expression levels of CD31, CD36, Egr-3, ILK, SDF-1α and Ephrin-B2 genes were consistently higher than in ATDPCs (9.9, 4.5, 20.4, 21.4, 29.5 and 1.4 times, respectively), the contrary was true for CD34 and vWF genes (0.1 and 0.5 times, respectively) (**Table S1**). In contrast, activation of VEGF and HIF-1α genes was equivalent in UCBMSC- and ATDPC-seeded implants. Importantly, there was no amplification of mouse mRNAs with any of the human FAM-labelled primers used in these experiments (data not shown).

We then used a fluorescent angiography procedure to determine the presence of functional microcirculatory vessels within cell-seeded implants. This method, based on the injection of a high molecular weight FITC-dextran through the tail lateral vein of live animals, permits to visualize vascular structures, including those within the implants and connected to the animal circulatory system. Analysis of the FITC-dextran-perfused animals revealed a functionally connected network that was better organized within Matrigel implants seeded with UCBMSCs than in those seeded with ATDPCs (**Figure 4C**). Merged fluorescence images showed human (mRFP1^+^) UCBMSCs in close physical association with morphologically mature microcirculatory vessels (**video** **S1**), while the corresponding ATDPCs were forming more disperse or disorganized structures.

### Implantation of an UCBMSC-seeded Fibrin Patch Reduced Infarct Size and Promoted Revascularization of Infarcted Myocardium

**Figure 5:**
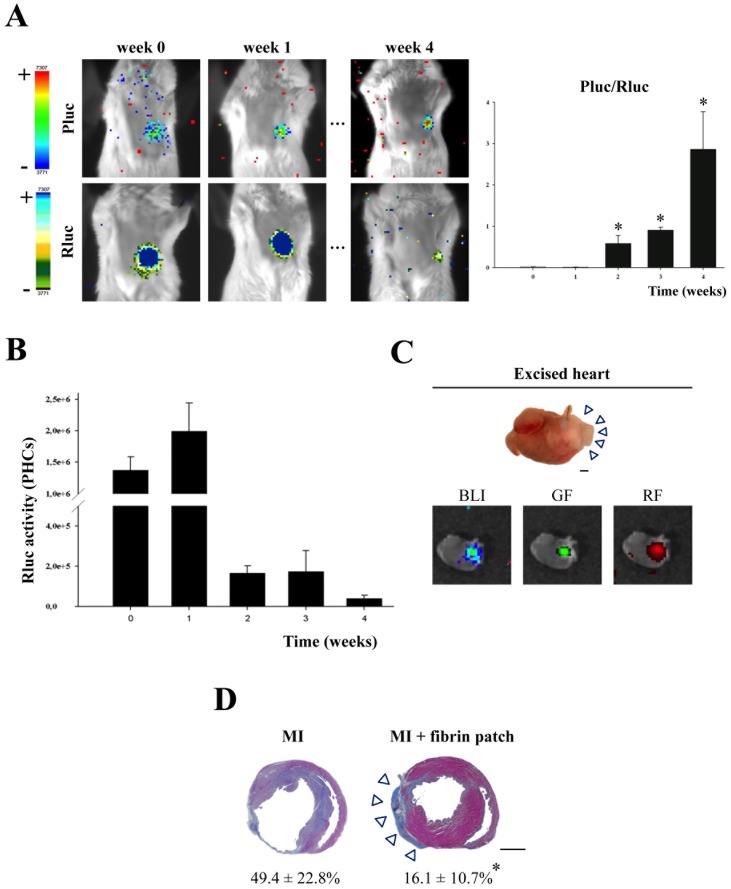
Reduction of infarct size by implantation of an UCBMSC-embedded fibrin patch. A ) Representative BLI images from dual labelled UCBMSCs within an implanted fibrin patch. Images of luciferase activity are also superimposed on b&w dorsal images of the recipient animal. Colour bars illustrate relative light intensities from Pluc and Rluc; low: blue and black; high: red and blue, respectively. Histograms represent BLI data expressed as the ratio between Pluc and Rluc signals. *P<0.05. **B**) Histogram represents over time monitoring of recorded Rluc PHCs following activation of CMVp, directly related to implanted cell survival, in MI-treated animals. N = 4. **C**) Representative photographs of a heart excised from a fibrin patch-treated animal showing bioluminescence, as well as green-(GF) and red-(RF) fluorescence emission. **D**) Representative sections from control and fibrin patch-treated MI-hearts following Masson’s Trichrome staining displaying changes in the infarcted area. Arrowheads point to the fibrin patch laid over infarcted myocardium; numeric values indicate % of scar area. *P = 0.001 and bars = 1 mm.

The response of UCBMSCs to ischemia was examined in a mouse MI model generated by ligation of the proximal left anterior descending (LAD) coronary artery according to a previously-described procedure [25]. Thus, uninduced UCBMSCs co-transduced with CD31p-Pluc-eGFP and CMVp-Rluc-mRFP1 were delivered over the infarct area within a fibrin patch. BLI analysis showed that the ratio between CD31p-regulated Pluc and CMVp-regulated Rluc activities significantly increased over time (*P*<0.05) (**Figure 5A**). We also observed an increase of ∼33% in the recorded CMVp-regulated Rluc activity during the first week after cell transplantation (**Figure 5B**), indicating that cell number increased after cell delivery followed by a decrease in Rluc activity reaching ∼3.3% of the initial, four weeks post-implantation (**Figure 5B**). Whole hearts excised from MI-animals bearing an UCBMSC-embedded fibrin patch displayed bioluminescence, as well as red- and green-fluorescence emission from implanted cells, confirming the activation of both reporters at implantation sites (**Figure 5C**).

**Figure 6:**
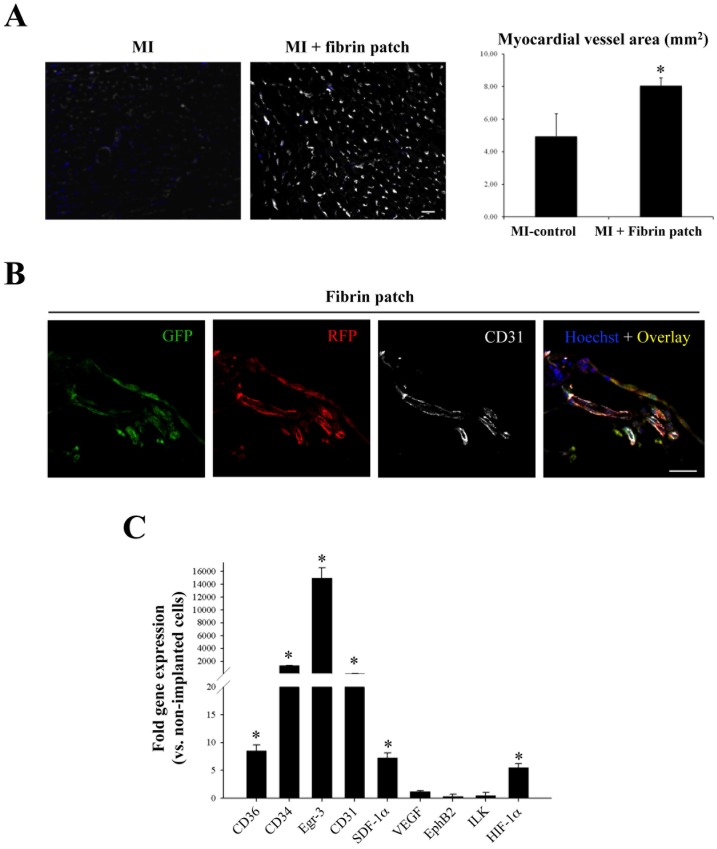
Increased revascularization of infarcted myocardium following fibrin patch delivery. **A**) Representative images showing staining of vascular system with biotinylated GSLI B4 isolectin in myocardium tissue sections from a MI-control and a fibrin patch-treated MI-heart. Histogram shows the percentage of vessel area in both groups. *P = 0.014 and bar = 25 µm. **B**) View of UCBMSCs within the fibrin patch 4 weeks post-implantation. Fluorescence images from a representative cell-embedded patch showing UCBMSCs expressing, from left to right, CD31p-regulated eGFP, CMVp-regulated mRFP1, CD31 protein stained using a specific antibody, and the overlay image of the 3 previous channels together with Hoechst nuclei staining. Bar = 25 µm. **C**) qRT-PCR analysis of human gene transcription within the fibrin patch 4 weeks post-implantation. N = 4 and *P<0.05.

Subsequent histological analysis of myocardium slices demonstrated a larger amount of salvaged myocardial tissue in fibrin patch treated-MI animals in comparison with control-MI animals without fibrin patches. This observation was corroborated by morphometric analysis of heart serial sections that showed a significant reduction in LV infarct area in the infarcted hearts that had received an UCBMSC-embedded fibrin patch compared to untreated MI-controls (16.1±10.7 vs. 49.4±22.8%; *P* = 0.001) (**Figure 5D**). Further analysis revealed additional changes induced following implantation of UCBMSC-embedded fibrin patches. By using specific staining with biotinylated GSLI B4 isolectin, we evidenced that the infarct region subjacent to the fibrin patch had a significantly higher vessel density in comparison with untreated MI-controls (8.1±0.6 vs. 4.9±1.9 mm^2^; *P* = 0.014) (**Figure 6A**). Nevertheless, no fluorescent human cells could be detected within subjacent myocardium (**Figure S5A**). Four weeks post-implantation, human mRFP1^+^and eGFP^+^cells within the fibrin patches laid over infarcted myocardium had self organized forming cell network structures and expressed CD31 protein (**Figure 6B**). QRT-PCR analysis also showed high activation of human Egr-3 (192-fold), CD34 (108-fold), CD31 (45-fold), CD36 (9.5-fold), SDF-1α (8-fold), and HIF-1α (5.5-fold) genes (**Figure 6C**). Moreover, *de novo* acquisition of cardiomyocyte-specific phenotypic traits by human UCBMSCs was analyzed. Following 4 weeks over infarcted myocardium, qRT-PCR and immunohistochemistry analysis showed the activation of human SERCA2 (12-fold), Cx-43 (7.3-fold), sarcomeric α-actinin (3.1-fold), and MEF2A (1.4-fold) genes (**Figure S5B**). However, no human mRFP1^+^cells expressing cardiac troponin I (cTnI) protein were detected within the fibrin patches (**Figure S5C**).

## Discussion

Adequate vascular precursors for regenerative purposes are still in great need. Delivery of pro-angiogenic cytokines has been tested with modest benefits [26], [1], and the potential of endothelial progenitor cells [27], bone marrow MSCs [10], embryonic and induced pluripotent stem cells is currently under preclinical scrutiny [28], [12].

Herein, we first show that UCBMSCs can be induced *in vitro* to acquire expression of new cell markers, increased Ac-LDL uptake and migratory behaviour, as well as the capacity to form well-organized Matrigel-mediated cell networks and to activate circulating pro-angiogenic myeloid cells. We cannot categorically refer to the induced cells as endothelial cells due to the use of a marker expression pattern alone which not allow us to distinguish them from other cells with overlapping phenotype, Ac-LDL uptake, and GSLI B4 staining [27]. In addition, induced cells did not show the characteristic cobblestone morphology. Nevertheless, the acquisition of angiogenic and vasculogenic properties, including increased cell migration, self organization into well-developed cell networks and activation of accessory pro-angiogenic myeloid cells, by UCBMSCs could be anticipated from our *in vitro* experiments.

Since initial description of putative endothelial progenitors by Asahara *et al* [29], distinct cell populations with varying phenotype, vascular homeostasis contribution and purity have been studied [27]. First described progenitors contained a mixture of hematopoietic CD45^+^and CD45^−^ cell types, including the circulatory myeloid-lineage cells used in our *in vitro* chemotaxis and co-culture assays [21], [30], [31]. Collectively, these cell populations, which have differentially been termed circulating angiogenic cells, hemangiocytes or accessory (supportive) mononuclear myeloid cells [32]–[34], are mobilized from bone marrow in response to systemic VEGF levels contributing to vascular repair within damaged tissues. Recently, a novel hierarchy of genuine, high proliferative vascular precursors has been identified [35], [36]. Regarding the activation of circulating myeloid cells, we found that these cells were induced to migrate in response to the conditioned medium from induced UCBMSCs in a SDF-1α-dependent manner, as it was shown by abolishing migratory capacity with a specific SDF-1α antibody. Furthermore, tested angiogenic-supportive myeloid (CD45^+^) cells were seen to be closely associated with the linear cell networks generated by induced UCBMSCs, again pointing to a robust vasculogenic-inducing activity by UCBMSCs following culture in EGM-2. These results, together with the finding that SDF-1α mediates neovascularization *in vivo* [37], suggested that *in vivo* transplantation of UCBMSCs could promote an active recruitment of circulating pro-angiogenic cells that, in turn, could support vascular growth.

Our *in vitro* findings were then corroborated in two distinct *in vivo* models using the same uninduced UCBMSCs that *in vitro* had demonstrated similar properties to those of angiogenic cells. By using uninduced UCBMSCs, we also avoided potential pitfalls due to proliferation stress following growth in EGM-2. First, we observed that, following subcutaneous co-injection of a mixture of UCBMSCs and Matrigel in mice, these cells also had pro-angiogenic activity *in vivo*. In these experiments, we used a dual reporter labelling strategy with two luciferases: one regulated by the CMVp and constitutively expressed (used as an internal control of cell number) and a second one under the control of the human CD31p (a frequently used reporter of vascular cell differentiation). This approach allowed us to use the ratio of the light produced by the CD31p-regulated Pluc relative to that produced by the CMVp-regulated Rluc to evaluate level of cell differentiation independently of the number of cells. This is relevant for several reasons; cells have difficulties surviving long periods within the implants due to the pro-apoptotic effect of Matrigel [38] and cells transduced with CD31p-Pluc-eGFP show a basal level of CD31p activation that needed to be taken into consideration. Although we do not know for certain the reason for this spurious expression, we speculate that the CD31p is leaky, allowing a certain level of expression even under conditions where cell differentiation has not taken place. The alternative explanation for this background based on the existence of a small fraction of pre-induced cells was not supported by further analyses of eGFP and CD31 expression in pre-implanted UCBMSCs. Thus, our results show that cells subcutaneously co-injected with Matrigel in mice expressed over time increasing levels of CD31, as was measured by the increase in the ratio between Pluc and Rluc signals relative to that initially recorded. Imaging data was independently validated by qRT-PCR that showed increased activation of CD34, CD36, vWF, CD31, Egr-3, Ephrin-B2, ILK, SDF-1α, VEGF, and HIF-1α genes.

Confocal microscope analysis of surgically removed implants showed that implanted cells were closely associated with well-organized structures functionally connected to the host vascular system. In both regards CD31p-regulated reporter activity and association with new functional microvasculature, UCBMSCs appeared more efficient than equivalently labelled ATDPCs. Moreover, the *in vivo* angiogenic response exhibited by UCBMSCs was more robust than that observed *in vitro* suggesting that implanted cells may be subject to host stimuli that inhibit the apoptotic progression characteristic of terminally differentiated cells in Matrigel [38]. We also demonstrate that UCBMSCs associated with structures that were filled with the FITC-dextran injected through the tail vein of the host and were therefore hollow and well connected to the host vascular system, in contrast with the branched structures lacking luminal space previously observed in the standard tests *in vitro*. However, we could not unequivocally determine whether implanted cells were in direct contact with the vascular lumen or had endothelial function. We speculate that the strong human ILK and SDF-1α gene expression found within UCBMSC-seeded implants may promote the incorporation of host vascular progenitors into the newly created vessels resulting in the observed integrative behaviour. This approach could be used to address still unresolved issues with potential impact in regenerative medicine, including the possibility of generating human vascular implants *ex vivo*; such as the relative contribution of implanted human and host vascular cells to the newly formed vasculature, or how hybrid vessels grow and connect.

A mouse MI model was finally used to examine the behaviour of UCBMSCs in response to ischemia. Thus, we implanted an UCBMSC-seeded fibrin patch over the ischemic area, a procedure that enhances retention of applied cells allowing them to exert the therapeutic benefits during at least 4 weeks post-implantation. To date, bone marrow monocytes, myoblasts and adult MSCs have been administered into the ischemic heart at various time-points and by different routes (intracoronary delivery, direct myocardial injection and endomyocardial implantation by catheters) with modest benefits, mainly due to limited implanted cell engraftment and survival in the fibrous myocardium [39]–[42]. This has motivated the exploration in recent years of novel regeneration strategies such as those based in tissue engineering approaches combining cells, biomaterials and growth factors [43]–[47]. Nevertheless, the increase in engraftment and retention of delivered cells within the infarcted myocardium continues been the key challenge for current protocols of cardiac regeneration.

A similar fibrin patch-based cell delivery system was previously used to co-implant endothelial cells generated from human embryonic stem cells and smooth muscle cells following MI in both mice and pigs [13]. In the current work, the analysis of MI-treated animals implanted with a human UCBMSC-embedded patch showed markedly reduction in fibrous scar area presumably due to increased neovascularization induced by implanted cells in a paracrine manner, as Xiong and co-workers also suggested [13]. In contrast with the report of Xiong *et al*, we herein implanted uninduced cells, avoiding unnecessary previous *in vitro* induction. Although no cells from the patch could be found into underlying myocardium, we show that UCBMSCs applied over the infarcted myocardium were induced to express CD31 and self organized into cell networks within the patch, as observed by BLI and histological analysis respectively, likely in response to factors raised from subjacent damaged tissue. Moreover, the elevated level of human SDF-1α gene expression detected in UCBMSC-embedded fibrin patches could activate the attraction of recipient vascular progenitors to subjacent myocardium increasing vessel growth and blood supply. Concurrent with this, subjacent myocardium contained a significantly larger number of blood vessels in UCBMSC-embedded fibrin patch-treated MI hearts. Thus, we hypothesize that reinforcing feedback loops might be established between ischemic myocardium and the patch generating transcriptional changes in human UCBMSCs (as detected by qRT-PCR), and subsequent myocardial revascularization and tissue salvage. Signals from infarcted myocardium also appear to activate certain cardiomyocyte-specific phenotypic traits in implanted UCBMSCs. However, no induction of cTnI expression was detected. Collectively, these observations not only point to the therapeutic potential of UCBMSCs, but also suggest strategies to analyze the existing interactions between implanted therapeutic cells and damaged tissues.

In summary, taken together, the presented results show that UCBMSCs can be induced *in vitro* to acquire angiogenic and vasculogenic properties and contribute to vascular growth *in vivo*. These cells, implanted using a fibrin patch to enhance cell retention, increase revascularization of infarcted myocardium. Factors released from implanted cells are suggested as significant contributors to cell therapeutic efficacy. UCBMSCs are a valuable model to further analyze the mechanisms that instruct multipotent stem cells to gain vascular cell characteristics and a promising cell source for vascular growth and tissue repair. Our results also emphasize the need for procedures that improve UCBMSC delivery, migration, and survival towards injured myocardium.

## Materials and Methods

### Cell Culture

Isolation and culture of UCBMSCs were previously described in detail [48], [49]. Cells were maintained in α-MEM (Sigma) supplemented with 10% fetal bovine serum (FBS), 1 mM L-glutamine and 1% penicillin/streptomycin (Invitrogen). Cells were differentiated in complete EGM-2 medium (Lonza) for 15 days.

Primary adult human skin fibroblasts and HUVECs were purchased from Inbiomed (Inbiobank) and Lonza, respectively. Human CPCs and ATDPCs were correspondingly isolated from peripheral venous blood and subcutaneous fat as described in [21], [50].

Written informed consent was obtained from all subjects, the study was approved by the University hospital Germans Trias i Pujol Clinical Research Ethics Committee and the study protocols conformed to the principles outlined in the Declaration of Helsinki.

### *In vitro* Migration Assay

Migration capacity was analyzed using Culture-Inserts (Ibidi). Cells (3 10^4^/well) were seeded and grown to confluence. When the two wells were filled with adherent cells, a cell-free gap of 400 µ m was created by removal of the Culture-Inserts. Migratory cells were tracked by phase-contrast microscopy and the percentage of surface recovering index was quantified using the Image J software (NIH).

### Matrigel Assay

Cell network formation was assessed using the *In vitro Angiogenesis Assay Kit* (Chemicon). Cells were detached with non-enzymatic cell dissociation medium (Sigma) to avoid cell membrane antigen proteolysis and seeded (1×10^5^) onto 24-well plates pre-coated with 200 µl ECMatrix (Matrigel). Generated networks were examined after 5 h in 10 images captured at a constant magnification (100×) and angiogenesis capacity was then calculated as the number of network circles per mm^2^, an equivalent measurement to classic network length determination [51]. N = 3 per tested condition.

### Transwell Chemotaxis Assay

Circulating myeloid cells (1×10^5^ cells/100 µl) were seeded in the upper chamber of 6.5 mm Transwells (Corning Life Sciences) having 5 µm-pore size polycarbonate membrane inserts separating a lower chamber containing 600 µl of each migration medium. After a 5 h incubation period, cells that had migrated to the lower chamber were counted. For the study using SDF-1α-blocking antibody, conditioned medium (CM) from EGM-2-cultured cells was pre-incubated with 20 µg/ml anti-human SDF-1α (Santa Cruz Biotech.) for 30 min at 37°C before its addition to the lower chamber of the wells. Medium supplemented with 2% FBS and 150 ng/ml human recombinant SDF-1α (R&D Systems) was used as positive control.

### Vector Constructs and Lentiviral Transduction

UCBMSCs and ATDPCs were co-transduced (2×10^6^ transduction units/ml, MOI = 21, 48 h) with the following lentiviral vectors: 1) CMVp-Rluc-mRFP1 containing a chimeric construct of the Rluc reporter gene and mRFP1 in a PHR lentiviral vector, under the CMVp transcriptional control [52]; and 2) CD31p-Pluc-eGFP, a fusion reporter vector comprising Pluc and eGFP under the transcriptional control of the 0,25 KB NorI/PstI fragment of human CD31p, which showed a higher transcriptional activity in ECs in comparison with monocytic cells [53]. Cells were selected for mRFP1 expression by using FACS.

### Animal Studies

Animal studies were approved by the Animal Experimentation Unit Ethical Committee of the Catalan Institute of Cardiovascular Sciences (ICCC) and complies with guidelines concerning the use of animals in research and teaching as defined by the Guide For the Care and Use of Laboratory Animals (NIH Publication No. 80-23, revised 1996).

### Matrigel Plug Assay

Four SCID mice (Charles River Laboratories) were anesthetized by intraperitoneal injection of 100 mg/kg ketamine (Merial) plus 3.3 mg/kg xilacine (Henry Schein). A mixture of 100 µl Matrigel with 3×10^5^ UCBMSCs or ATDPCs suspended in 100 µl of PBS was subcutaneously injected in the paraspinal space of the mouse (4 independent injection sites per animal, 2 injected animals and 8 injection sites per cell type) using a 21-25G size needle. CD31p activation was monitored by increase in luciferase activity using BLI [50]. Mice were intraperitonaly injected with 150 µl of luciferin (Pluc substrate) (16.7 mg/ml in physiological serum) (Braun) or through the tail lateral vein with 25 µl of benzyl coelenterazine (Rluc substrate) (1 mg/ml in 50% v/v propileneglycol/ethanol) (Nanolight Technology). Mice were placed under the ORCA-2BT Imaging System (Hamamatsu Photonics) and Pluc and Rluc images acquired during 5 min and 5 sec, respectively. Mice were monitored during 5 days. Image analysis was performed using the Wasabi software (Hamamatsu Photonics). Recorded light fluxes were expressed as photon counts (PHCs) after discounting background using the formula PHCs = (total number of PHCs in the area of interest)-[(number of pixels in the area of interest)*(background average PHCs/pixel)].

### Fluorescence Angiography

Before sacrifice, the high molecular weight fluorescent tracer for microcirculatory vessels FITC-dextran (10 mg/ml, Sigma) was injected through animal tail lateral vein. Animals were anesthetized with O_2_/isoflurane (2%) and hearts were arrested in diastole with 68.4 mM NaCl, 59 mM KCl, 11.1 mM Glucose, 1.9 mM NaHCO3, 29.7 mM 2,3-butanedione monoxime and 1000 U Heparin. Matrigel implants were removed and inspected by confocal microscopy or used for RNA extraction and gene expression analysis.

### Myocardial Infarction Model and Fibrin-based Cell Delivery

Eight female SCID mice (Charles River Laboratories) were anesthetized with a mixture of O_2_/isoflurane (2%) (Baxter), and mechanically ventilated (90 breath/min, tidal volume 0.1 ml) (TOPO dual mode ventilator, Kent Scientific Corporation). Surgical anesthesia plane was confirmed by visual examination of breathing level and by response to gentle stimulus, i.e. toe/foot squeeze. An anterior thoracotomy was performed and the proximal LAD coronary artery was occluded using an intramural stitch (7–0 silk suture).

Eight µl of Tissucol Duo fibrin adhesive solution (Baxter) was mixed with either 1.5×10^6^ lentiviral transduced UCBMSCs or the same volume of saline solution and jellified with 8 µl of trombin solution (Baxter). After the ligation, fibrin patch was placed over the infarcted myocardium using Glubran® 2 (Cardiolink) to seal the edges of the patch to the healthy myocardium. Mice were monitored by BLI during four weeks and sacrificed as previously described. Hearts were excised, analyzed by BLI, green and red fluorescence emission, and processed for gene expression, morphometric and histological analysis.

### Statistical Analysis

Statistical analysis was performed using a two-tailed Student’s t test. Values are expressed as mean ± SD. One-way ANOVA with Tukey B post-hoc analysis was applied to determine significance among more than 2 groups. Descriptive statistics were performed using SPSS Statistics (15.0.1 version, SPSS Inc.). Statistical tests were considered significant when *P*<0.05.

## Supporting Information

Figure S1 **Baseline and acquired characteristics by primary human UCBMSC cultures. A**) Characterization of the basic traits exhibited by UCBMSCs. Histogram shows fluorescence intensity data, expressed as mean ± SD, from surface antigen expression analysis by flow cytometry. A.U. = arbritary units. Cells (over 95%) were homogenously, consistently positive for CD105, CD44, CD166, CD29 and CD90, as well as negative for CD117, CD106, CD34, CD45, CD14, VEGF-R2 and CD133. Standard MSC pluripotency was also demonstrated by specifically cell differentiation into adipogenic, chondrogenic and osteogenic lineages. Images show differentiated (upper row) and control (bottom row) cell cultures following staining with, from left to right, Oil red O, Alizarin red S, and Alcian blue. Respective negative controls are also shown. **B**) Flow cytometry analysis of CD34 and CD31 expression in EGM-2-induced UCBMSCs. HMVECs-dBI cells were used as positive controls.
(TIF)

Figure S2 **Analysis of cell proliferation and migration.** **A**) Cell growth curves from UCBMSCs in control and EGM-2 and conditions. *P = 0.009. Specific detection of the cell proliferation marker, Ki67 by indirect immunofluorescence is also shown. A minimum of 10 microscopic fields per condition and experiment (N = 3) were analyzed. Bars = 20 µm. Graph representing cell growth curves of UCBMSCs in both conditions. Data were also from three independent experiments performed in duplicate. **B**) Representative images of the *in vitro* tracking of migratory cells in Culture-Inserts. Adult human skin fibroblasts were used as positive migratory cells. Bars = 400 µm. Histogram represents quantitative differences in the surface recovery capacity exhibited by tested cells. N = 3, *P<0.001 and ^†^ *P* = 0.004.
(TIFF)

Figure S3 **BLI monitoring of lentivirally co-transduced UCBMSCs cultured in EGM-2. A**) UCBMSCs emitting light derived from Rluc activity plated at a range of concentration from 50 to 5,000 cells/well. BLI analysis showed highest light intensity at 5000 cells/well density. Rluc emission correlated to cell number is also plotted. N = 3 **B**) Representative images showing light emission from Pluc and Rluc activity from an induced UCBMSC culture. Recorded Pluc/Rluc during cell induction in EGM-2 is also shown. N = 3 and *P<0.001.
(TIF)

Figure S4 **Analysis of mRFP1, eGFP and CD31 expression in co-transduced UCBMSCs.** Representative confocal images showing mRFP1^+^(red), eGFP^+^(green) and mRFP1^+^/eGFP^+^cells in control and EGM-2-induced dual lentivirally co-transduced UCBMSCs prior cell implantation (**A**) and following 5 days within a subcutaneous Matrigel implant (**B**). Specific detection of CD31 (violet) by EGM-2-induced mRFP1^+^/eGFP^+^cells is also shown. Note that, in *in vitro* control conditions, the amount of mRFP1^+^/eGFP^+^and eGFP^+^cells were undetectable while, at animal sacrifice, the proportion of GFP^+^cells that were not also mRFP1^+^was extremely low. Five days post-injection, all mRFP1^+^cells were usually eGFP^+^. Nuclei are counterstained with Hoescht (blue). A minimum of 15 microscopic fields were analyzed. Bars = 20 and 40 µm in A and B panels, respectively.
(TIF)

Figure S5 **Analysis of UCBMSC migration into subjacent infarcted myocardium and acquisition of cardiomyocyte-specific phenotypic traits. A**) Three representative overlay images showing distribution of UCBMSCs within fibrin patch attached above the infarcted myocardium 4 weeks post-implantation. Constitutive mRFP1 (red), inducible eGFP (green), and both human and mouse CD31 (white) expression, as well as Hoescht nuclei counterstaining (blue) are shown. Fibrin patch and subjacent infarcted myocardium (My) appear limited by the yellow dotted line. Note that there was not human cell migration into infarcted myocardium. A minimum of 10 microscopic fields were analyzed by confocal microscopy. Bar = 25 µm. **B**) QRT-PCR analysis of human cardiac-specific gene transcription within the fibrin patch 4 weeks post-implantation. N = 4 and *P<0.05. **C**) Representative overlay image illustrating large amounts of UCBMSCs within fibrin patch fixed 4 weeks over the infarcted myocardium (My). Expression of mRFP1 (red) and mouse cTnI (white) are detected. Nuclei are counterstained with Hoescht (blue). Note that, in these experiments, expression of eGFP was not examined and there was not acquisition of cTnI protein by implanted cells. A minimum of 10 microscopic fields were analyzed by confocal microscopy. Bar = 75 µm.
(TIF)

Method S1 (DOCX)

Method S2 (DOCX)

Method S3 (DOCX)

Method S4 (DOCX)

Method S5 (DOCX)

Method S6 (DOCX)

Method S7 (DOCX)

Method S8 (DOCX)

Method S9 (DOCX)

Table S1 Analysis of gene activation within cell-seeded Matrigel plugs. ^*^Average Ct values from at least three independent experiments performed in duplicate were used to calculate fold changes in gene expression (2^−ΔΔCt^) using GAPDH as reference.
(DOCX)

Video S1 Participation of UCBMSCs in the formation of functional microvascular structures in a Matrigel co-implantation model in mice. Representative three-dimensional reconstruction of a histological section derived from a surgically-removed human UCBMSC-seeded Matrigel implant, in which the organization of human cells forming a functionally connected network with the host circulatory system can be clearly observed.
(AVI)
