# Supplementary figures and images for: Human Umbilical Cord Blood-Derived Mesenchymal Stem Cells Promote Vascular Growth In Vivo
Source: PLoS One. 2012 Nov 16;7(11):e49447. doi: 10.1371/journal.pone.0049447 (PMC3500294; doi:10.1371/journal.pone.0049447)

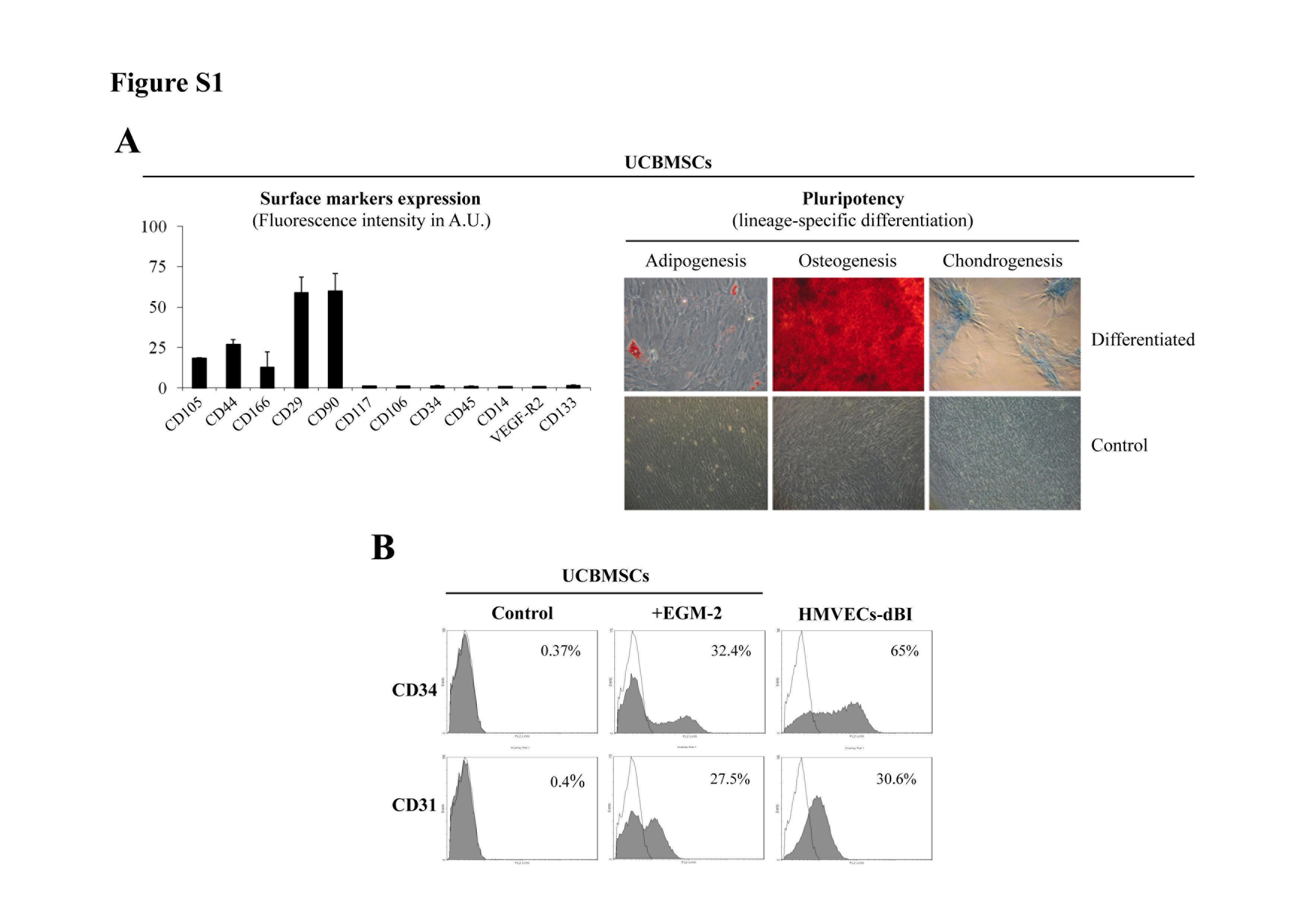

Supplement: Figure S1 — Baseline and acquired characteristics by primary human UCBMSC cultures. A) Characterization of the basic traits exhibited by UCBMSCs. Histogram shows fluorescence intensity data, expressed as mean ± SD, from surface antigen expression analysis by flow cytometry. A.U. = arbritary units. Cells (over 95%) were homogenously, consistently positive for CD105, CD44, CD166, CD29 and CD90, as well as negative for CD117, CD106, CD34, CD45, CD14, VEGF-R2 and CD133. Standard MSC pluripotency was also demonstrated by specifically cell differentiation into adipogenic, chondrogenic and osteogenic lineages. Images show differentiated (upper row) and control (bottom row) cell cultures following staining with, from left to right, Oil red O, Alizarin red S, and Alcian blue. Respective negative controls are also shown. B) Flow cytometry analysis of CD34 and CD31 expression in EGM-2-induced UCBMSCs. HMVECs-dBI cells were used as positive controls. (TIF) [file pone.0049447.s001.tif]

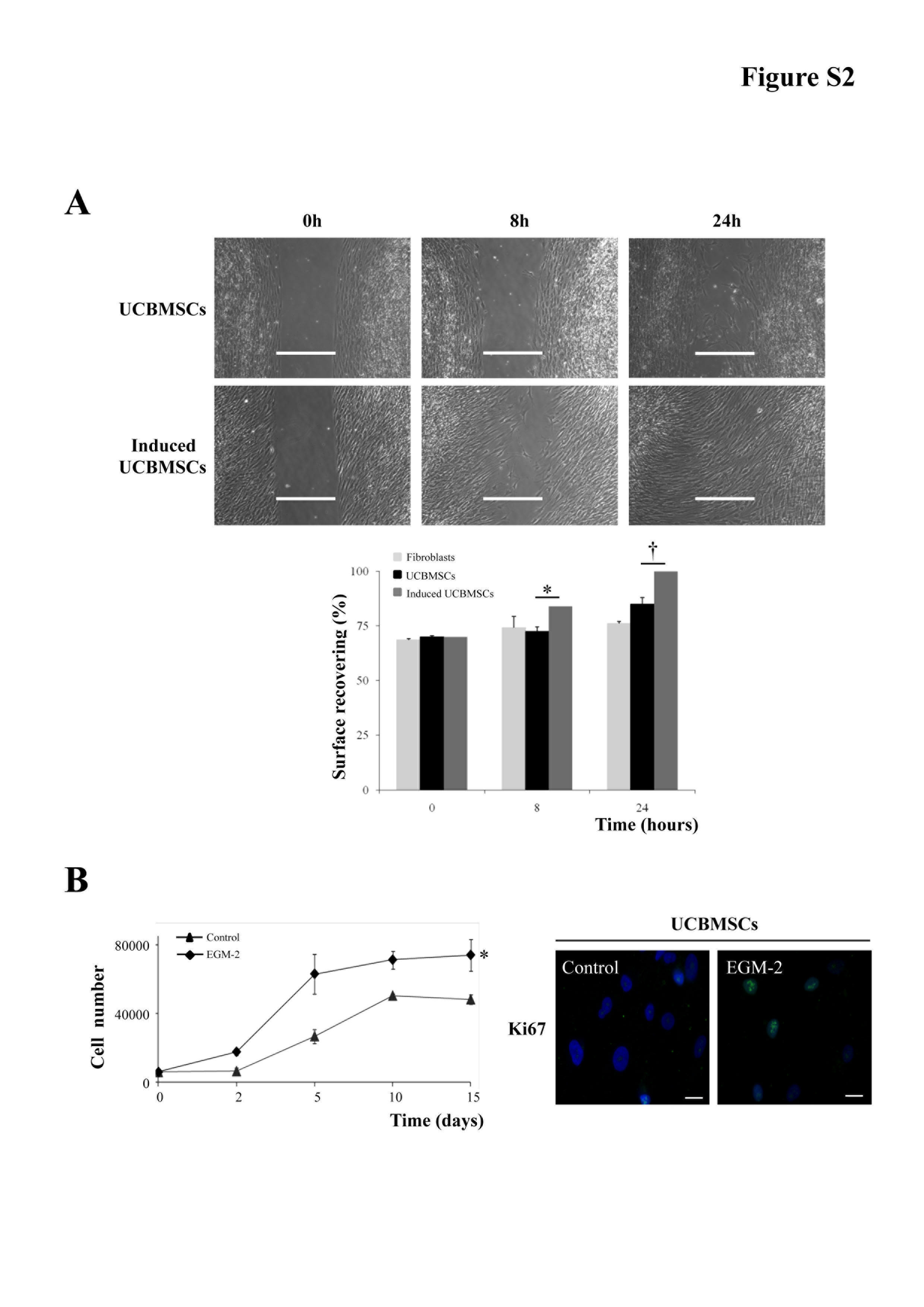

Supplement: Figure S2 — Analysis of cell proliferation and migration. A) Cell growth curves from UCBMSCs in control and EGM-2 and conditions. *P = 0.009. Specific detection of the cell proliferation marker, Ki67 by indirect immunofluorescence is also shown. A minimum of 10 microscopic fields per condition and experiment (N = 3) were analyzed. Bars = 20 µm. Graph representing cell growth curves of UCBMSCs in both conditions. Data were also from three independent experiments performed in duplicate. B) Representative images of the in vitro tracking of migratory cells in Culture-Inserts. Adult human skin fibroblasts were used as positive migratory cells. Bars = 400 µm. Histogram represents quantitative differences in the surface recovery capacity exhibited by tested cells. N = 3, *P<0.001 and † P = 0.004. (TIFF) [file pone.0049447.s002.tiff]

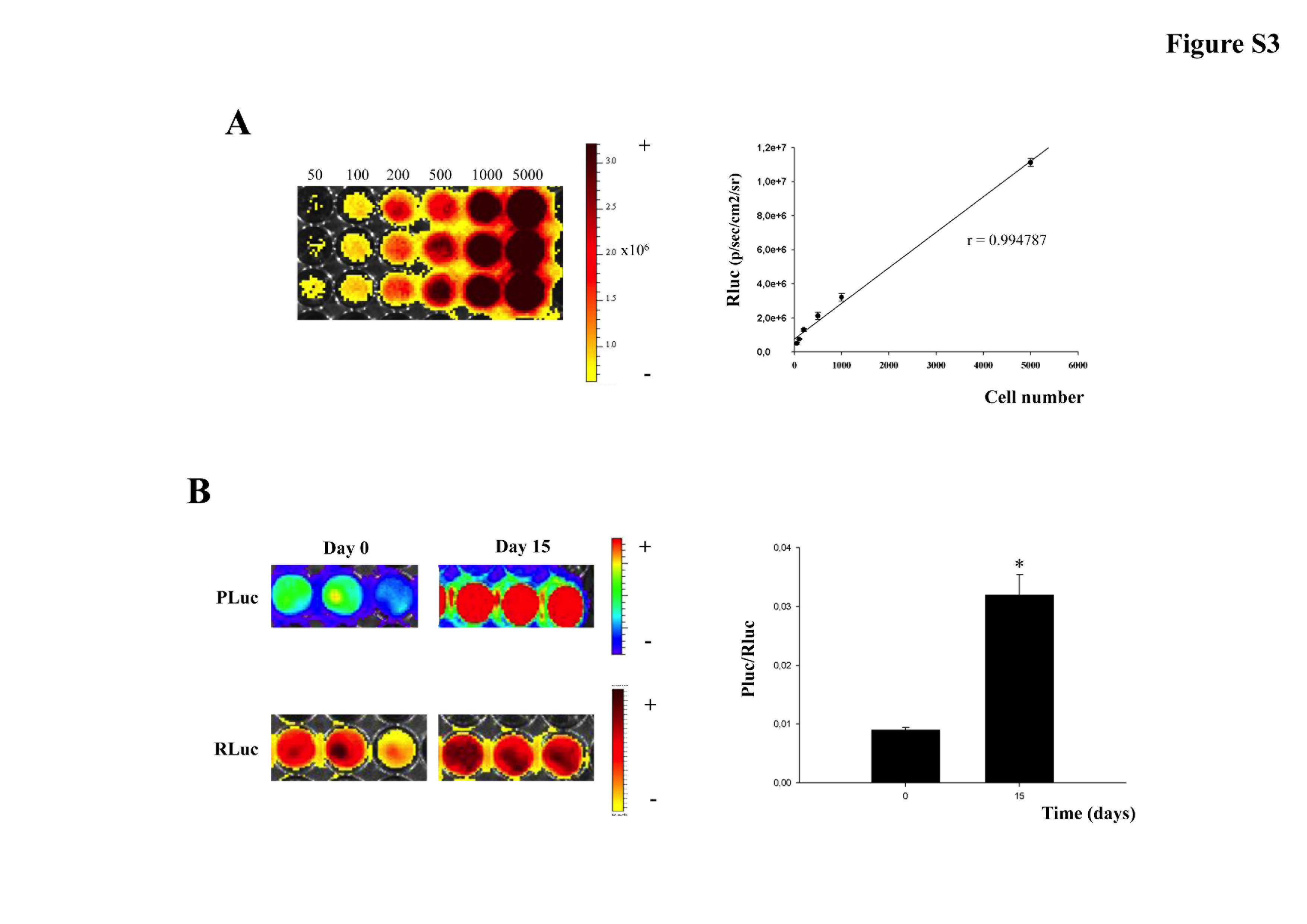

Supplement: Figure S3 — BLI monitoring of lentivirally co-transduced UCBMSCs cultured in EGM-2. A) UCBMSCs emitting light derived from Rluc activity plated at a range of concentration from 50 to 5,000 cells/well. BLI analysis showed highest light intensity at 5000 cells/well density. Rluc emission correlated to cell number is also plotted. N = 3 B) Representative images showing light emission from Pluc and Rluc activity from an induced UCBMSC culture. Recorded Pluc/Rluc during cell induction in EGM-2 is also shown. N = 3 and *P<0.001. (TIF) [file pone.0049447.s003.tif]

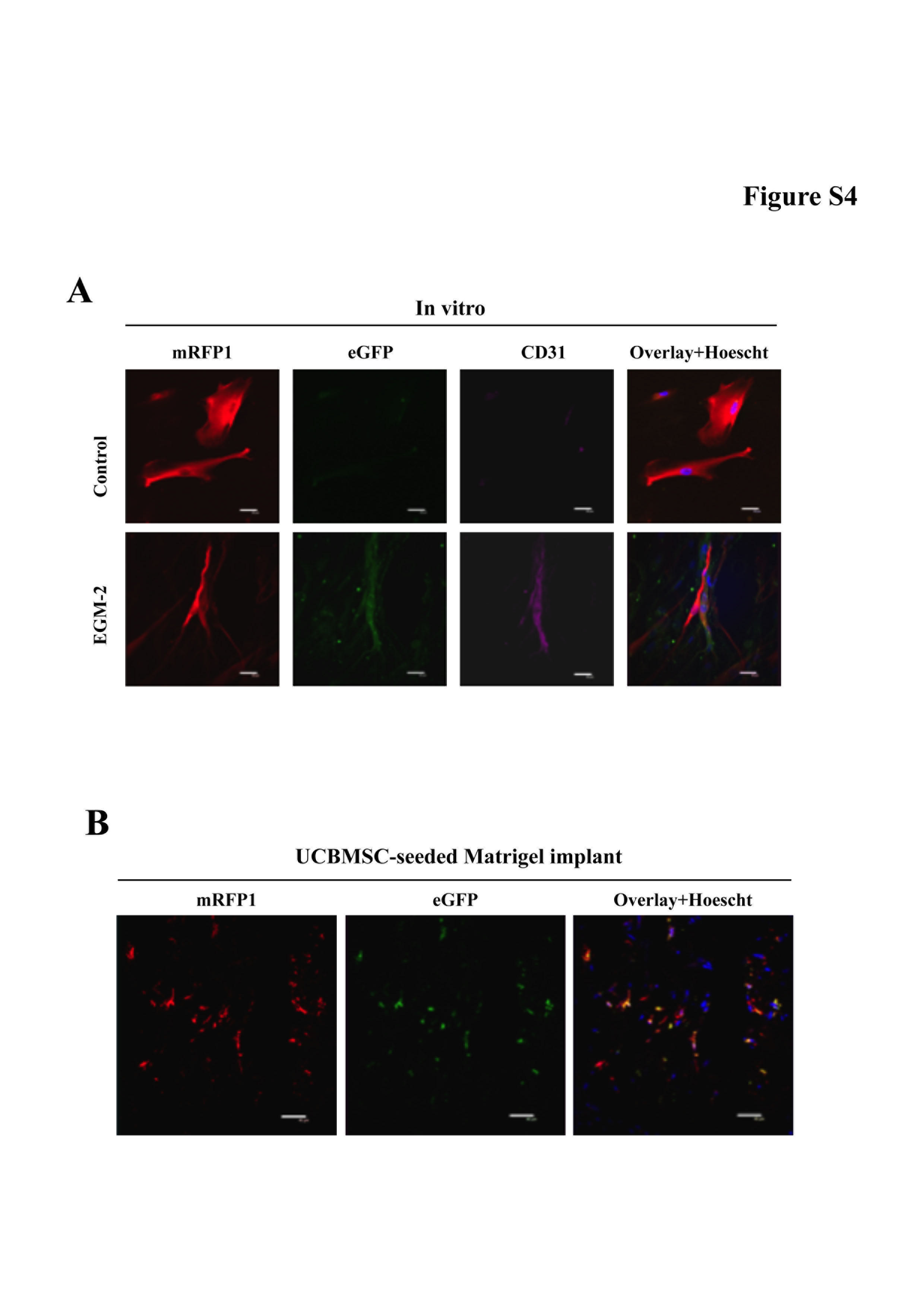

Supplement: Figure S4 — Analysis of mRFP1, eGFP and CD31 expression in co-transduced UCBMSCs. Representative confocal images showing mRFP1+(red), eGFP+(green) and mRFP1+/eGFP+cells in control and EGM-2-induced dual lentivirally co-transduced UCBMSCs prior cell implantation (A) and following 5 days within a subcutaneous Matrigel implant (B). Specific detection of CD31 (violet) by EGM-2-induced mRFP1+/eGFP+cells is also shown. Note that, in in vitro control conditions, the amount of mRFP1+/eGFP+and eGFP+cells were undetectable while, at animal sacrifice, the proportion of GFP+cells that were not also mRFP1+was extremely low. Five days post-injection, all mRFP1+cells were usually eGFP+. Nuclei are counterstained with Hoescht (blue). A minimum of 15 microscopic fields were analyzed. Bars = 20 and 40 µm in A and B panels, respectively. (TIF) [file pone.0049447.s004.tif]

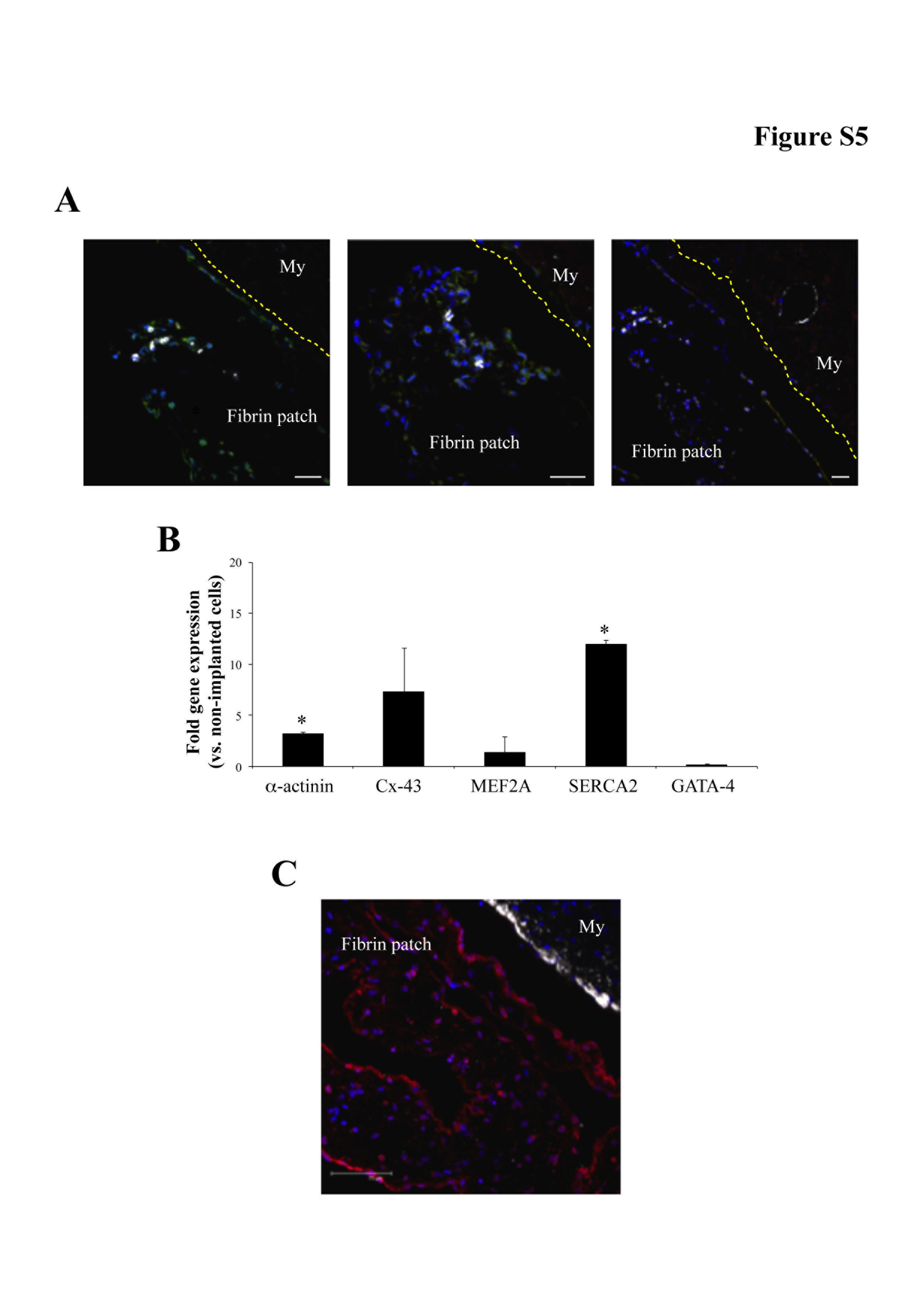

Supplement: Figure S5 — Analysis of UCBMSC migration into subjacent infarcted myocardium and acquisition of cardiomyocyte-specific phenotypic traits. A) Three representative overlay images showing distribution of UCBMSCs within fibrin patch attached above the infarcted myocardium 4 weeks post-implantation. Constitutive mRFP1 (red), inducible eGFP (green), and both human and mouse CD31 (white) expression, as well as Hoescht nuclei counterstaining (blue) are shown. Fibrin patch and subjacent infarcted myocardium (My) appear limited by the yellow dotted line. Note that there was not human cell migration into infarcted myocardium. A minimum of 10 microscopic fields were analyzed by confocal microscopy. Bar = 25 µm. B) QRT-PCR analysis of human cardiac-specific gene transcription within the fibrin patch 4 weeks post-implantation. N = 4 and *P<0.05. C) Representative overlay image illustrating large amounts of UCBMSCs within fibrin patch fixed 4 weeks over the infarcted myocardium (My). Expression of mRFP1 (red) and mouse cTnI (white) are detected. Nuclei are counterstained with Hoescht (blue). Note that, in these experiments, expression of eGFP was not examined and there was not acquisition of cTnI protein by implanted cells. A minimum of 10 microscopic fields were analyzed by confocal microscopy. Bar = 75 µm. (TIF) [file pone.0049447.s005.tif]
