# Supplementary material for: Human Umbilical Cord Blood-Derived Mesenchymal Stem Cells Promote Vascular Growth In Vivo
Source: PLoS One. 2012 Nov 16;7(11):e49447. doi: 10.1371/journal.pone.0049447 (PMC3500294; doi:10.1371/journal.pone.0049447)
Supplement: Method S1 — (DOCX) [file pone.0049447.s006.docx]

**Method S1**

###### **Cell culture.** The study originally included 25 umbilical cord blood (UCB) samples that were analyzed for their capacity to generate primary cultures of mesenchymal stem cells. Four primary cultures were generated demonstrating a low selection yield, also reported by previous authors [1-3]. After plating of mononuclear cells, a few elongated fibroblast-like cells attached to the plastic culture dishes and rapidly expanded 4 weeks post-extraction.

# Endothelial growth medium (EGM)-2 comprised endothelial basal medium (EBM)-2 supplemented with SingleQuots Bullet-Kit [0.5 ng/ml VEGF, 5 ng/ml EGF, 10 ng/ml bFGF, 20 ng/ml long R3-IGF-1, 22.5 μg/ml heparin, 1 μg/ml ascorbic acid, 0.2 μg/ml hydrocortisone, gentamicin (1/1000 dilution) and 2% fetal bovine serum (FBS)], all purchased from Lonza.

Human dermal blood microvascular cells (HMVECs-dBI) were also obtained from Lonza and maintained in EGM-2 until they were used as positive controls in flow cytometry experiments.

Cell cultures were usually maintained under standard culture conditions, with media replacement every three days.

**Supplemental references**

1. Bieback K, Kern S, Klüter H, Eichler H (2004) Critical parameters for the isolation of mesenchymal stem cells from umbilical cord blood. Stem Cells 22: 625-634.
2. Kern S, Eichler H, Stoeve J, Klüter H, Bieback K (2006) Comparative analysis of mesenchymal stem cells from bone marrow, umbilical cord blood, or adipose tissue. Stem Cells 24: 1294-1301.
3. Manca MF, Zwart I, Beo J, Palasingham R, Jen LS, et al. (2008) Characterization of mesenchymal stromal cells derived from full-term umbilical cord blood. Cytotherapy 10: 54-68.
