# Supplementary material for: Human Umbilical Cord Blood-Derived Mesenchymal Stem Cells Promote Vascular Growth In Vivo
Source: PLoS One. 2012 Nov 16;7(11):e49447. doi: 10.1371/journal.pone.0049447 (PMC3500294; doi:10.1371/journal.pone.0049447)
Supplement: Method S2 — (DOCX) [file pone.0049447.s007.docx]

**Method S2**

**qRT-PCR.** Total RNA was isolated from samples using the QuickPrep Extraction Kit (Amersham). Random hexamers and the Script^TM^ One-Step RT-PCR Kit (Bio-rad) were used to obtain cDNA from 2 μg of RNA. 2 μl of cDNA were amplified in a final volume of 50 μl containing 25 μl TaqMan 2X Universal PCR Master Mix, and 2 μl of FAM-labelled primer/probes purchased from Applied Biosystems: CD31 (Hs00169777_m1), CD34 (Hs00990732_m1), CD36 (Hs00169627_m1), CD45 (Hs00236304_m1), CD102 (Hs00168384_m1), von Willebrand Factor (vWF) (Hs00169795_m1), early growth response factor (Egr)-3 (Hs00231780_m1), SDF-1α (Hs00171022_m1), Ephrin-B2 (Hs00970627_m1), ILK (Hs00177914_m1), VEGF (Hs00173626_m1), SRF (Hs00182371_m1), sarcomeric α-actinin (Hs00241650_m1), GATA-4 (Hs00171403_m1), SERCA-2 (Hs00544877_m1), MEF2A (Hs0150409_m1), Cx-43 (Hs00748445_s1), and GAPDH (Hs99999905_m1). To detect HIF-1α, the PCR reaction contained primers: 5’-CTAGCCGAGGAAGAACTATGAACAT-3’ (forward) and 5’-CTGAGGTTGGTTACTGTTGGTATCA-3’ (reverse), and the FAM-labelled probe AAGGTATTGCACTGCACAGGCCACA (Applied Biosystems). Each sample was run in duplicate, and amplification data analyzed on the ABI Prism 7000 Sequence Detection System. The Δ threshold cycle (Ct) method was used to quantify relative expression for each gene using GAPDH as endogenous reference [1].

**Supplemental references**

1. Pfaffl M (2001) A new mathematical model for relative quantification in real-time RT-PCR. Nucleic Acids Res 29:e45.
