# Supplementary material for: Human Umbilical Cord Blood-Derived Mesenchymal Stem Cells Promote Vascular Growth In Vivo
Source: PLoS One. 2012 Nov 16;7(11):e49447. doi: 10.1371/journal.pone.0049447 (PMC3500294; doi:10.1371/journal.pone.0049447)
Supplement: Method S3 — (DOCX) [file pone.0049447.s008.docx]

**Method S3**

###### **Flow cytometry.** To measure expression of surface antigens, cells (5x10^5^/100 μl) were labeled with 10 μl of fluorescein- or phycoerythrin-conjugated monoclonal antibodies against human CD105 (Serotec), CD90, CD166, CD29, CD44, CD14, CD117, CD34, CD45, CD34 (BD Pharmingen), CD31 (Abcam Inc.), and CD133 and VEGFR-2 (R&D Systems) during 30 min at room temperature. Labelled isotype-matched IgG (Caltag Laboratories) were used as negative controls. Intensity levels for each antigen were calculated as the ratio between specific antibody and control (1 = no difference). Data acquisition and further analysis were carried out using a Coulter EPICS XL flow cytometer and the Expo32 software (Beckman Coulter), respectively.
