# Supplementary material for: Human Umbilical Cord Blood-Derived Mesenchymal Stem Cells Promote Vascular Growth In Vivo
Source: PLoS One. 2012 Nov 16;7(11):e49447. doi: 10.1371/journal.pone.0049447 (PMC3500294; doi:10.1371/journal.pone.0049447)
Supplement: Method S4 — (DOCX) [file pone.0049447.s009.docx]

**Method S4**

**Differentiation assays.** Adipogenic induction: Cells were cultured in α-MEM (Sigma) supplemented with 10% FBS, 1 mM L-glutamine and 1% penicillin/streptomycin (Invitrogen), 1 μM dexamethasone, 0.5 mM isobutylmethylxanthine, 10 μg/ml insulin and 100 μM indomethacin (Sigma). Medium was replaced every 3-4 days and, after a 14 day period, differentiated cells were detected following Oil red O (Sigma) staining that detects the presence of intracellular lipid accumulation. Osteogenic induction: Cells were cultured in α-MEM supplemented with 10% FBS, 1 mM L-glutamine and 1% penicillin/streptomycin, 100 nM dexamethasone, 10 mM sodium β-glycerophosphate, and 0.05 mM ascorbic acid during 14 days. Deposition of calcium matrix was then detected by staining with Alizarin red S (Sigma). Chondrogenic induction: Cells were differentiated using the Stem Pro Chondrogenesis Differentiation kit (Invitrogen). Inducing medium was continuously added to the cell culture during a 21 day period. Staining with Alcian blue (Sigma) was performed to visualize differentiated cells.
