# Supplementary material for: Human Umbilical Cord Blood-Derived Mesenchymal Stem Cells Promote Vascular Growth In Vivo
Source: PLoS One. 2012 Nov 16;7(11):e49447. doi: 10.1371/journal.pone.0049447 (PMC3500294; doi:10.1371/journal.pone.0049447)
Supplement: Method S5 — (DOCX) [file pone.0049447.s010.docx]

**Method S5**

**Indirect immunofluorescence.** Phenotypic characterization of control (uninduced), EGM-2-induced and lentivirally co-transduced cells, as well as Matrigel-mediated cell networks and circulating angiogenic-supportive myeloid cells were performed following fixation with 4% paraformaldehyde (Sigma) for 10 min at room temperature (RT). Subsequently, cells were permeabilized with magnesium-free PBS supplemented with 10% horse serum and 0.5% Triton X-100 (Sigma) during 2 h at RT, but cell surface antigens were detected without cell permeabilization. Specific monoclonal antibodies (mAbs) against human Ki-67, Egr-3, CD31 (2 μg/ml) (Santa Cruz Biotech.), HIF-1α (20 μg/ml), SM22α (6 μg/ml) (Abcam), VEGFR-2 (5 μg/ml; R&D Systems), CD34 and vWF (5 μg/ml) (BD Biosciences) were applied. When indicated, staining with biotinylated *Griffonia simplicifolia* Lectin I (GSLI) B4 isolectin (0.5 μg/ml; Vector Labs) was performed according to the manufacturer’s instructions. Cell nuclei were commonly stained with Hoechst (0.5 μg/ml) (Sigma) for 10 min at RT. Finally, secondary antibodies conjugated to Alexa Fluor 488, Streptavidin Alexa Fluor 488 (1 μg/ml), Alexa Fluor 568 (5 μg/ml) (Invitrogen), and Cy2 and Cy3 (1/1000 dilution) (Jackson Immunoresearch) were used to detect labelled cells by confocal laser scanning microscope (TCS SP5, Leica). Both mRFP1 and eGFP expression in lentivirally co-transduced cell cultures were directly analyzed by confocal microscopy and quantification of positive cells were completed using the ImageJ analysis software (NIH).
