# Supplementary material for: Human Umbilical Cord Blood-Derived Mesenchymal Stem Cells Promote Vascular Growth In Vivo
Source: PLoS One. 2012 Nov 16;7(11):e49447. doi: 10.1371/journal.pone.0049447 (PMC3500294; doi:10.1371/journal.pone.0049447)
Supplement: Method S6 — (DOCX) [file pone.0049447.s011.docx]

**Method S6**

**Western blotting.** Whole cell and cytoplasmic/nuclear extracts were generated using the Mammalian Cell Lysis kit and the CelLytic™ NuCLEAR™ Extraction Kit (Sigma) according to manufacturer´s instructions, respectively. Equivalent amounts of protein (assayed with the BCA protein assay; Pierce Biotech.) were fractionated by 10%-15% SDS-PAGE and transferred to nitrocellulose membranes (0.45 μm pore size; Bio-rad).

For CD31 detection, membranes were blocked in PBS supplemented with 5% BSA and 0.1% Tween-20 overnight at 4ºC, and, subsequently, a rabbit polyclonal antibody anti-CD31 (1 μg/ml; Santa Cruz Biotech.) was added for 1 h at room temperature (RT). A secondary polyclonal goat anti-rabbit immunoglobulins, coupled to horseradish peroxidase (HRP) (1/2000 dilution; DakoCytomation), was finally incubated. Three washes in PBS supplemented with 0.1% Tween-20 for 15 min at RT were performed between steps.

To detect β-actin, α-tubulin, Egr-3, VEGFR-2, VE-cadherin and Histone 3, membranes were blocked with PBS supplemented with 5% non-fat dry milk and 0.05% Tween-20 overnight at 4ºC and proved using the following specific antibodies: anti-β-actin (1/1000 dilution), anti-α-tubulin (1/500 dilution) (Sigma), VEGFR-2 (1/1000 dilution; Cell Signaling), VE-cadherin (1/20 dilution; Abcam), anti-Egr-3 and anti-Histone 3 (1μg/ml).

Specific protein bands were detected using a HRP conjugated secondary goat anti-mouse IgG (H+L) (1/50000 dilution; Pierce Biotech.) or a goat anti-rabbit IgG (1/2000 dilution; DakoCytomation) antibody, and visualized with the SuperSignal West Pico Chemiluminiscent Substrate (Pierce Biotech.) under the Bio-Rad Molecular Imager Chemidoc^TM^ XRS+. Protein levels were quantified using the Quantity one 1-D Analysis software (Bio-rad) and expressed as arbitrary units of optical density. Commonly, background signals were subtracted from a membrane region close to the bands of interest. When indicated, densitometric values were normalized to β-actin in order to control for variation in protein loading.
