# Supplementary material for: Human Umbilical Cord Blood-Derived Mesenchymal Stem Cells Promote Vascular Growth In Vivo
Source: PLoS One. 2012 Nov 16;7(11):e49447. doi: 10.1371/journal.pone.0049447 (PMC3500294; doi:10.1371/journal.pone.0049447)
Supplement: Method S7 — (DOCX) [file pone.0049447.s012.docx]

**Method S7**

###### **Analysis of Ac-LDL uptake.** Control and EGM-2-induced cells (1,000 cells/cm^2^) were incubated with acetylated low-density lipoprotein (Ac-LDL) conjugated to Alexa488 (10 μg/ml) (Molecular Probes) for 6 h at 37ºC, fixed and examined under a confocal laser scanning microscope (TCS SP5, Leica).
