# Supplementary material for: Human Umbilical Cord Blood-Derived Mesenchymal Stem Cells Promote Vascular Growth In Vivo
Source: PLoS One. 2012 Nov 16;7(11):e49447. doi: 10.1371/journal.pone.0049447 (PMC3500294; doi:10.1371/journal.pone.0049447)
Supplement: Method S8 — (DOCX) [file pone.0049447.s013.docx]

**Method S8**

# Cell proliferation. Cells were seeded into six-well dishes in duplicate at 1,000 cells/cm^2^ density. At indicated time points, cells were washed with calcium- and magnesium-free PBS (Invitrogen), and collected by standard trypsinization. Viable cells were counted by the trypan blue exclusion method using a hemocytometer. Cell population doubling time (Td) was calculated during the log phase of culture growth curves as previously reported [1]. Proliferation data were plotted on a common log scale and fit by linear regression. Td values were determined using the formula: Td= Log2/K (days), where K is the slope of the regression line.

**Supplemental references**

1. Zhuang SH, Burnstein KL (1998) Antiproliferative effect of 1alpha, 25-dihydroxyvitamin D3 in human prostate cancer cell line LNCaP involves reduction of cyclin-dependent kinase 2 activity and persistent G1 accumulation. Endocrinology 139: 1197-1207.
