# Supplementary material for: Human Umbilical Cord Blood-Derived Mesenchymal Stem Cells Promote Vascular Growth In Vivo
Source: PLoS One. 2012 Nov 16;7(11):e49447. doi: 10.1371/journal.pone.0049447 (PMC3500294; doi:10.1371/journal.pone.0049447)
Supplement: Method S9 — (DOCX) [file pone.0049447.s014.docx]

**Methods S9**

**Morphometric, histological, and immunohistochemical examination of infarcted myocardium.** After 4 weeks post-implantation, hearts were arrested in diastole with arrest solution (68.4 mM NaCl, 59 mM KCl, 11.1 mM Glucose, 1.9 mM NaHCO3, 29.7 mM BDM (2,3-butanedione monoxime), 1000U Heparin), excised, fixed, cryopreserved in 30 % sucrose in PBS, embedded in OCT (Sakura Finetek Europe B.V.), and finally snap-frozen in liquid nitrogen-cooled isopentane. Tissue blocks were then stored at -80°C until sectioning.

Mouse hearts were transversally cut into two segments at the ligation level. Serial cryosections 10 μm thick (4 sections, spaced 200 μm apart) from the apex segment were stained with Masson’s trichrome (collagen: blue, myocardium: red) for measurements. Infarct size was measured as a percentage of mean scar area in the total left ventricular wall surface. All sections were blindly examined and photographed using a stereoscope (TL RCI, Leica). Immunostaining to detect CD31 and cTnI was performed on cell cultures or cryosections using specific monoclonal antibodies (1:50 dilution; Abcam). Vessel area was assessed in sections stained with biotinylated GSLI B4 isolectin (Vectors Labs). Sections were counterstained with Hoechst (Sigma) and analyzed by confocal microscopy. Quantitative morphometric and histological measurements were completed using the ImageJ analysis software (NIH).
