# Supplementary material for: Human Umbilical Cord Blood-Derived Mesenchymal Stem Cells Promote Vascular Growth In Vivo
Source: PLoS One. 2012 Nov 16;7(11):e49447. doi: 10.1371/journal.pone.0049447 (PMC3500294; doi:10.1371/journal.pone.0049447)
Supplement: Table S1 — Analysis of gene activation within cell-seeded Matrigel plugs. *Average Ct values from at least three independent experiments performed in duplicate were used to calculate fold changes in gene expression (2−ΔΔCt) using GAPDH as reference. (DOCX) [file pone.0049447.s015.docx]

**Table S1**

|  | ***Fold gene expression****  *(Matrigel implants vs. non-seeded cells)* | | ***Ratio*** |
| --- | --- | --- | --- |
| **Gene** | **UCBMSC-**  **seeded implants** | **ATDPC-**  **seeded implants** | **UCBMSC/ATDPC**  **implants** |
| *CD31* | 378 | 38.3 | 9.9 |
| *CD34* | 2 | 28.2 | 0.1 |
| *CD36* | 77 | 17 | 4.5 |
| *vWF* | 2 | 3.8 | 0.5 |
| *Egr-3* | 102 | 5 | 20.4 |
| *VEGF* | 81 | 89 | 0.9 |
| *SDF-1α* | 11.8 | 0.4 | 29.5 |
| *HIF-1α* | 50 | 60 | 0.8 |
| *ILK* | 23.5 | 1.1 | 21.4 |
| *Ephrin-B2* | 2.1 | 1.5 | 1.4 |
